# Extranodal Marginal Zone Lymphoma of the Floor of the Mouth: A Rare Entity With Management Challenges

**DOI:** 10.7759/cureus.117490

**Published:** 2026-10-05

**Authors:** Nourelhouda Mouhib, Berhili Soufiane, Souad Margoum, Ahmed Bensghier, Mohamed Moukhlissi, Loubna Mezouar

**Affiliations:** 1 Department of Radiation Oncology, Hassan II Oncology Center, Mohammed VI University Hospital, Faculty of Medicine and Pharmacy, Oujda, MAR; 2 Department of Radiation Oncology, Hassan II Oncology Center, Mohammed VI University Hospital, Oujda, MAR

**Keywords:** extranodal marginal zone lymphoma, floor of the mouth, malt lymphoma, oral cavity, radiotherapy

## Abstract

Extranodal marginal zone lymphoma of mucosa-associated lymphoid tissue (MALT lymphoma) involving the oral cavity is rare, and localization to the floor of the mouth is particularly uncommon. Its indolent course and nonspecific clinical presentation may lead to diagnostic and therapeutic challenges. Diagnosis relies on histopathological examination combined with immunohistochemical analysis to establish the B-cell phenotype and exclude other small B-cell lymphomas. We report the case of a 48-year-old man with stage IIE MALT lymphoma of the floor of the mouth associated with ipsilateral submandibular lymph node involvement. Given the large tumor volume, systemic therapy was initially favored. The patient received four cycles of rituximab monotherapy with an insufficient response. A repeat biopsy excluded histological transformation, and rituximab, cyclophosphamide, doxorubicin, vincristine, and prednisolone chemoimmunotherapy was subsequently administered, with persistent local disease. External radiotherapy was then delivered to the oral cavity and cervical lymph node regions (24 Gy), with a boost to the floor-of-mouth lesion to a total dose of 30 Gy. Follow-up positron emission tomography/computed tomography demonstrated a complete metabolic response, and the patient remained clinically free of recurrence 18 months after radiotherapy. This case highlights the diagnostic and therapeutic challenges of MALT lymphoma at an exceptional oral site and illustrates the importance of radiotherapy in achieving local disease control after an insufficient response to systemic therapy.

## Introduction

Extranodal marginal zone lymphoma of mucosa-associated lymphoid tissue (EMZL, or MALT lymphoma) is an indolent subtype of B-cell non-Hodgkin lymphomas characterized by a clonal proliferation of marginal zone B cells in extranodal tissues. Together with nodal and splenic marginal zone lymphomas, it is one of the three subtypes of marginal zone lymphoma, and it is the most frequent of the three [1]. It is frequently associated with chronic antigenic stimulation of infectious or autoimmune origin [2].

The most frequently involved extranodal sites include the stomach, salivary glands, thyroid, lungs, ocular adnexa, skin, and breast. In contrast, involvement of the head and neck region outside the salivary glands is uncommon [2,3]. B-cell non-Hodgkin lymphomas account for most lymphomas arising in this region, and only about 2% of them affect the oral cavity [3]. Primary EMZL of the oral cavity is therefore particularly rare, and the floor of the mouth is an exceptional site, with only a limited number of cases described in the literature [4-6].

Clinically, EMZL mainly affects adults, with a median age at diagnosis of approximately 60 to 65 years. Men and women are affected with similar overall incidence, but a female predominance is observed at certain sites, notably the salivary glands, where autoimmune diseases such as Sjögren’s syndrome are frequently associated [1,7,8]. Lymphomas of the oral cavity most often present as a local swelling with or without ulceration [8], and the disease typically remains confined to the tissue of origin for prolonged periods [2]. Nevertheless, local recurrence can occur, and, in rare cases, progression to systemic disease or transformation into diffuse large B-cell lymphoma has been reported [9].

MALT lymphomas of the oral cavity generally follow an indolent clinical course and have a favorable prognosis. However, diagnosis can be challenging because of the potential morphological and immunohistochemical overlap with reactive lymphoid proliferations and other small B-cell lymphomas. For localized EMZL, involved-site radiotherapy is generally considered a preferred treatment option, whereas systemic therapy may be required in selected clinical situations [10,11]. Nevertheless, the rarity of this presentation limits the available evidence regarding the optimal therapeutic approach [4].

We report a rare case of EMZL arising in the floor of the mouth. This unusual presentation highlights the diagnostic challenges associated with this anatomical site and the complexities of therapeutic decision- making in the absence of site-specific treatment recommendations.

## Case presentation

A 48-year-old man with a smoking history of seven pack-years and no notable past medical history presented with a painless swelling of the left floor of the mouth that had slowly increased in size over a three-year period. During the course of the disease, the lesion was associated with difficulty in mastication, without deterioration in his general condition. There were no clinical features or personal or family history suggestive of Sjögren’s syndrome or other autoimmune disease.

Clinical examination revealed a painless, smooth mass measuring approximately 5 cm in the left floor of the mouth, with no evidence of mandibular bone involvement. A 2-cm ipsilateral submandibular lymphadenopathy was also noted. Cervicofacial computed tomography (CT) demonstrated a left-sided lesion of the floor of the mouth measuring 52 × 27 mm, without bone lysis, along with enlargement of a submandibular lymph node (Figure 1).

**Figure 1:**
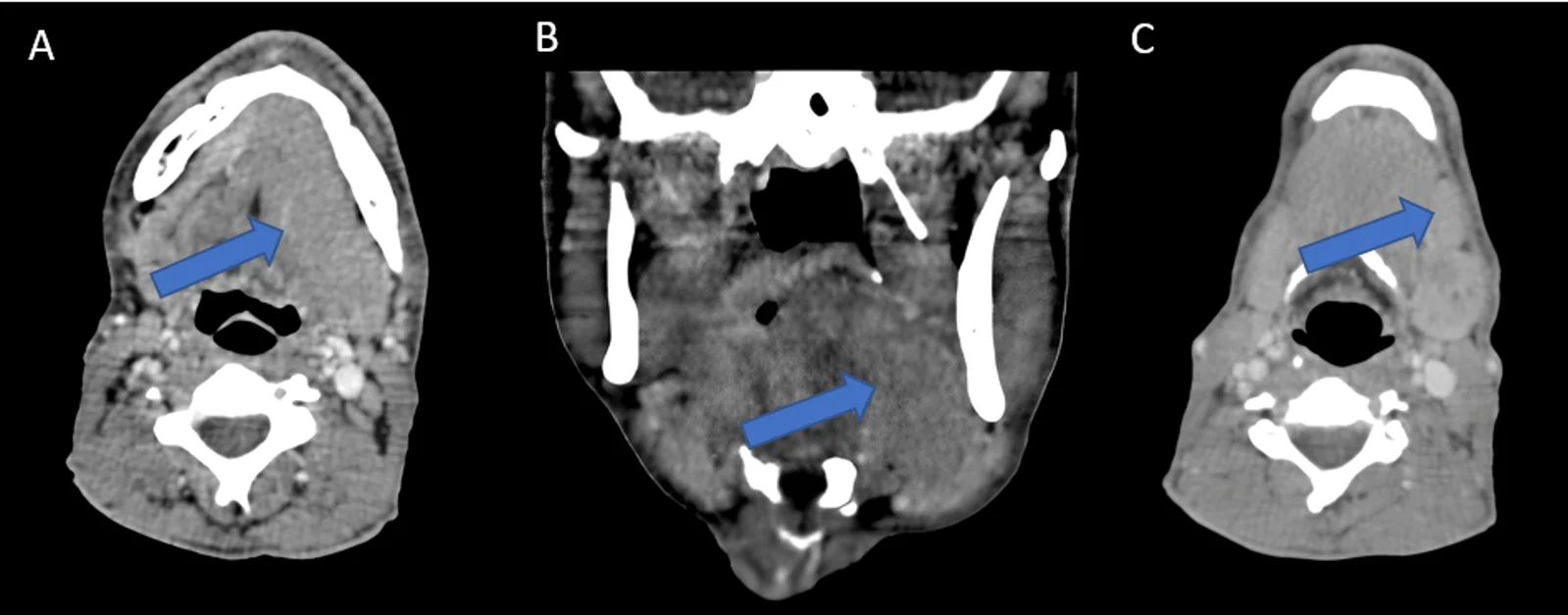
Pretreatment contrast-enhanced computed tomography (CT) images. (A) Axial CT image showing a left-sided floor-of-mouth lesion measuring 52 × 27 mm (arrow). (B) Coronal CT image showing the left-sided floor-of-mouth lesion (arrow). (C) Axial CT image showing an ipsilateral submandibular lymph node measuring 2 cm in its longest dimension (arrow).

Histopathological examination of the floor-of-mouth biopsy showed a diffuse proliferation of small to medium-sized lymphoid cells involving the lamina propria beneath the oral squamous epithelium. No salivary gland tissue was described in the biopsy specimen. Immunohistochemical analysis demonstrated strong and diffuse membranous expression of CD20 by the neoplastic cells, consistent with a B-cell phenotype, together with BCL2 expression. The neoplastic cells were negative for CD5, CD10, CD23, cyclin D1, and CD138. This immunophenotypic profile, in conjunction with the morphological findings, argued against the main differential diagnoses, including chronic lymphocytic leukemia/small lymphocytic lymphoma, mantle cell lymphoma, follicular lymphoma, and plasma cell neoplasm. CD21 staining did not reveal a significant follicular dendritic cell network. The Ki-67 proliferation index was low, at approximately 10%. Overall, the morphological and immunophenotypic findings supported the diagnosis of MALT lymphoma (Figure 2).

**Figure 2:**
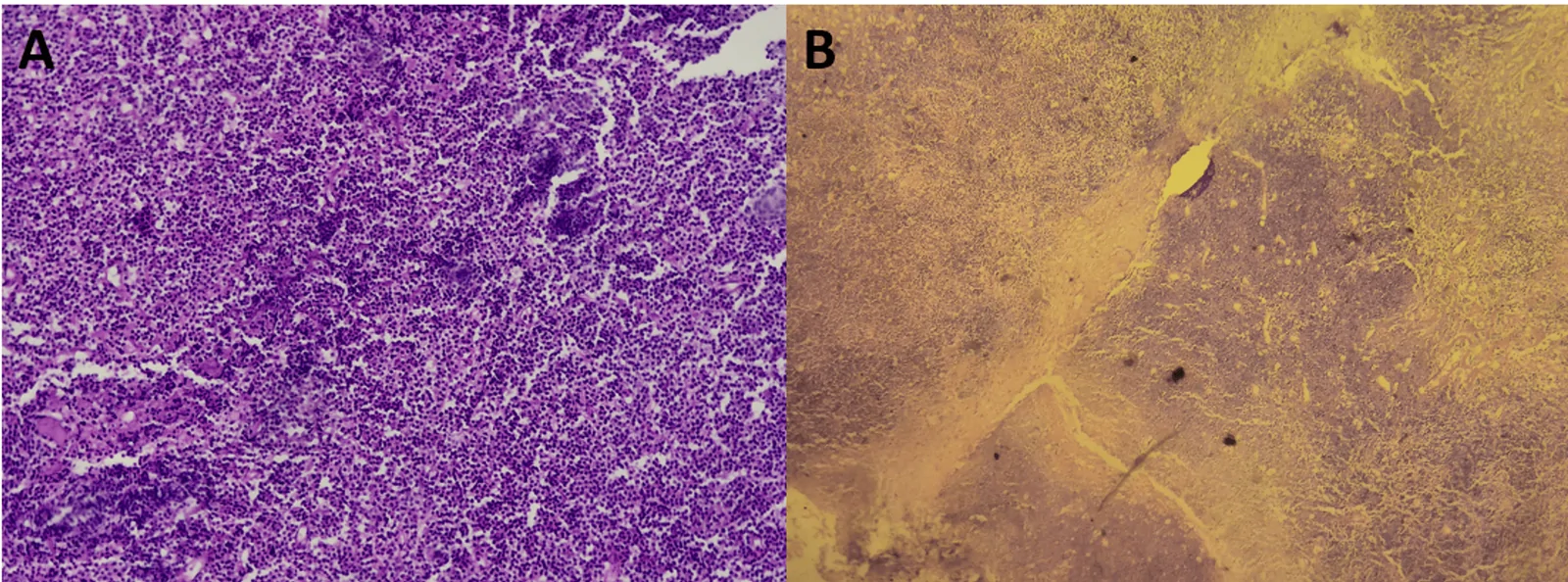
Histopathological and immunohistochemical findings. (A) Histological section showing a diffuse lymphomatous proliferation predominantly composed of small-sized lymphoid cells (hematoxylin and eosin, ×20). (B) Immunohistochemical staining for cluster of differentiation 20 showing strong and diffuse membranous expression in the neoplastic lymphoid cells.

A complete staging work-up, including thoraco-abdominopelvic CT scanning, bone marrow biopsy, and upper gastrointestinal endoscopy with systematic biopsies, revealed no additional sites of disease. Serological testing for hepatitis B and C viruses and human immunodeficiency virus (HIV) was negative. A pretreatment positron emission tomography/computed tomography (PET/CT) was not performed. The ipsilateral submandibular lymph node was considered involved on clinical and radiological grounds, based on its enlargement to approximately 2 cm on cervicofacial CT; no lymph node biopsy was performed.

Based on these findings, the lymphoma was clinically classified as stage IIE according to the modified Ann Arbor classification. The patient initially received four cycles of rituximab monotherapy. Response assessment by CT showed incomplete anatomical regression of the floor-of-mouth lesion, from 52 × 27 mm to 47 × 27 mm, while the ipsilateral submandibular lymph node remained stable. Given this limited response, a second biopsy of the floor-of-mouth lesion was performed three weeks after the last rituximab cycle to exclude histological transformation. It showed the same histological and immunophenotypic profile as the initial biopsy, with no evidence of transformation. He subsequently underwent six cycles of rituximab, cyclophosphamide, doxorubicin, vincristine, and prednisolone (R-CHOP) chemoimmunotherapy. Post-treatment CT showed further but incomplete regression of the floor-of-mouth lesion to 39 × 19 mm, while the ipsilateral submandibular lymph node remained stable. No additional disease sites were identified. Given the persistent residual disease after systemic therapy, radiotherapy was undertaken. At the time of referral to our radiotherapy department, the previously reported difficulty in mastication had resolved, despite the persistence of radiologically measurable disease.

Volumetric modulated arc therapy (VMAT) was subsequently delivered to the oral cavity and bilateral cervical nodal levels I to III at a dose of 24 Gy in 12 fractions. Apart from the ipsilateral submandibular lymph node, no additional cervical lymph nodes were radiologically suspicious; the remaining cervical nodal levels were treated electively. The residual gross tumor volume (GTV) encompassed the floor-of-mouth lesion and the adjacent ipsilateral submandibular lymph node, and the clinical target volume (CTV) was generated by adding a 1-cm margin to the GTV. This was followed by a 6-Gy boost in three fractions to the floor-of-mouth lesion and the ipsilateral submandibular lymph node, bringing the total dose to 30 Gy in 15 fractions at both involved sites ​​​​​​(Figure 3).

**Figure 3:**
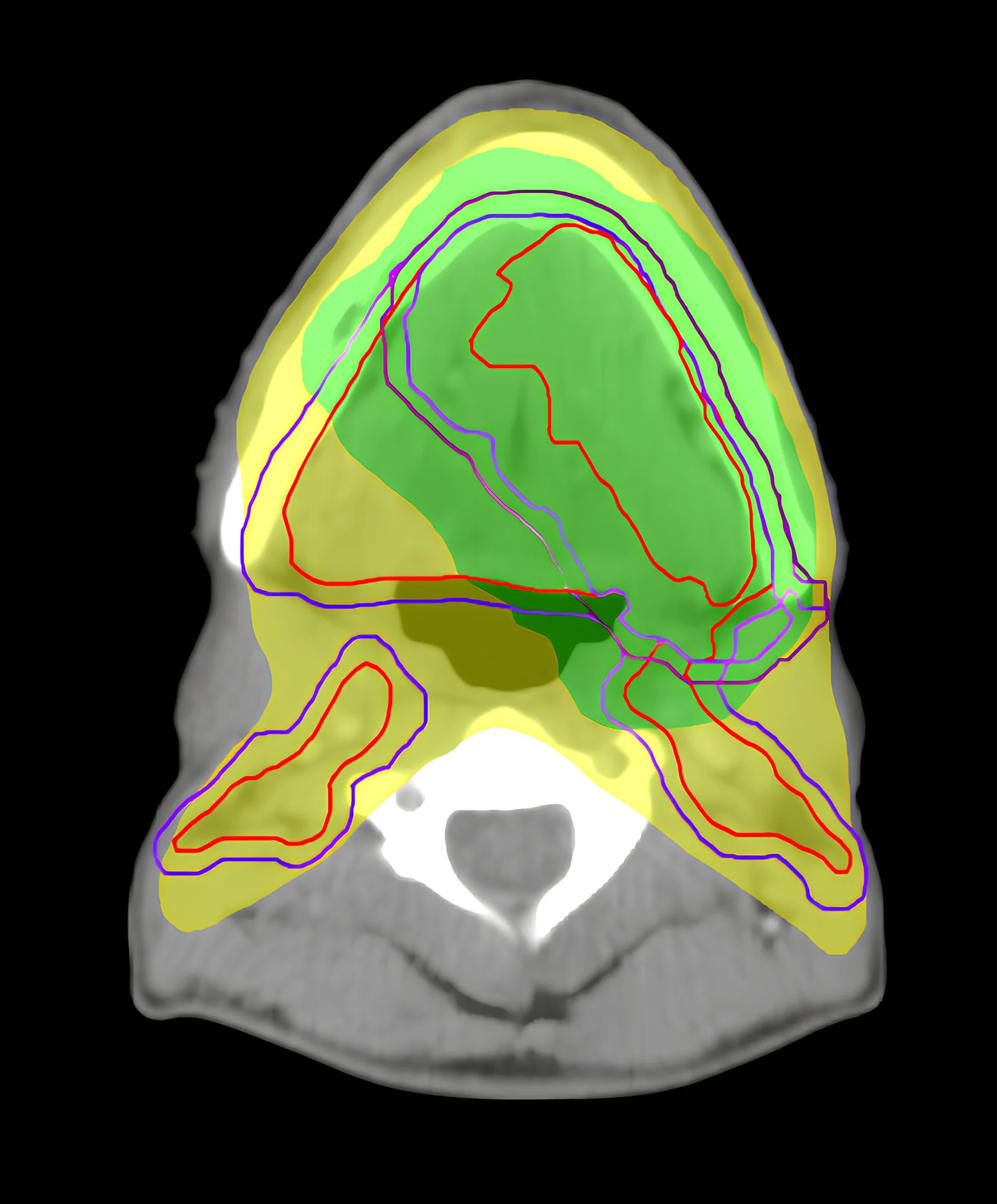
Radiotherapy dose distribution showing 24 Gy delivered to the oral cavity and bilateral cervical nodal levels I–III (yellow), followed by a boost to a total dose of 30 Gy to the floor-of-mouth lesion and the ipsilateral submandibular lymph node (green).

Treatment planning allowed adequate salivary gland and mandibular sparing, with mean doses of 15.84 Gy and 19.72 Gy to the right and left parotid glands, respectively, while the mandibular dose constraint was well respected. Acute toxicity was prospectively assessed through weekly clinical evaluations during radiotherapy. The patient experienced only mild transient dysphagia and minimal oropharyngeal erythema, consistent with grade 1 toxicity according to the Common Terminology Criteria for Adverse Events (CTCAE) version 5.0. These symptoms resolved rapidly without sequelae. Clinical examination three months after completion of radiotherapy showed a marked reduction in the size of the floor-of-mouth mass. At six months, cervico-thoraco-abdominopelvic CT demonstrated further regression of the floor-of-mouth lesion measuring 33 × 19 mm compared with 39 × 19 mm before radiotherapy. The ipsilateral submandibular lymph node initially measuring 2 cm had also decreased to approximately 1 cm. At nine months after radiotherapy, 18F-fluorodeoxyglucose (18F-FDG) PET/CT showed no suspicious hypermetabolic activity at the previously involved sites (Deauville score 2). Although this finding was consistent with a complete metabolic response, pretreatment FDG avidity had not been documented because no baseline PET/CT was performed; therefore, metabolic response could not be formally established by comparison with baseline imaging (Figure 4).

**Figure 4:**
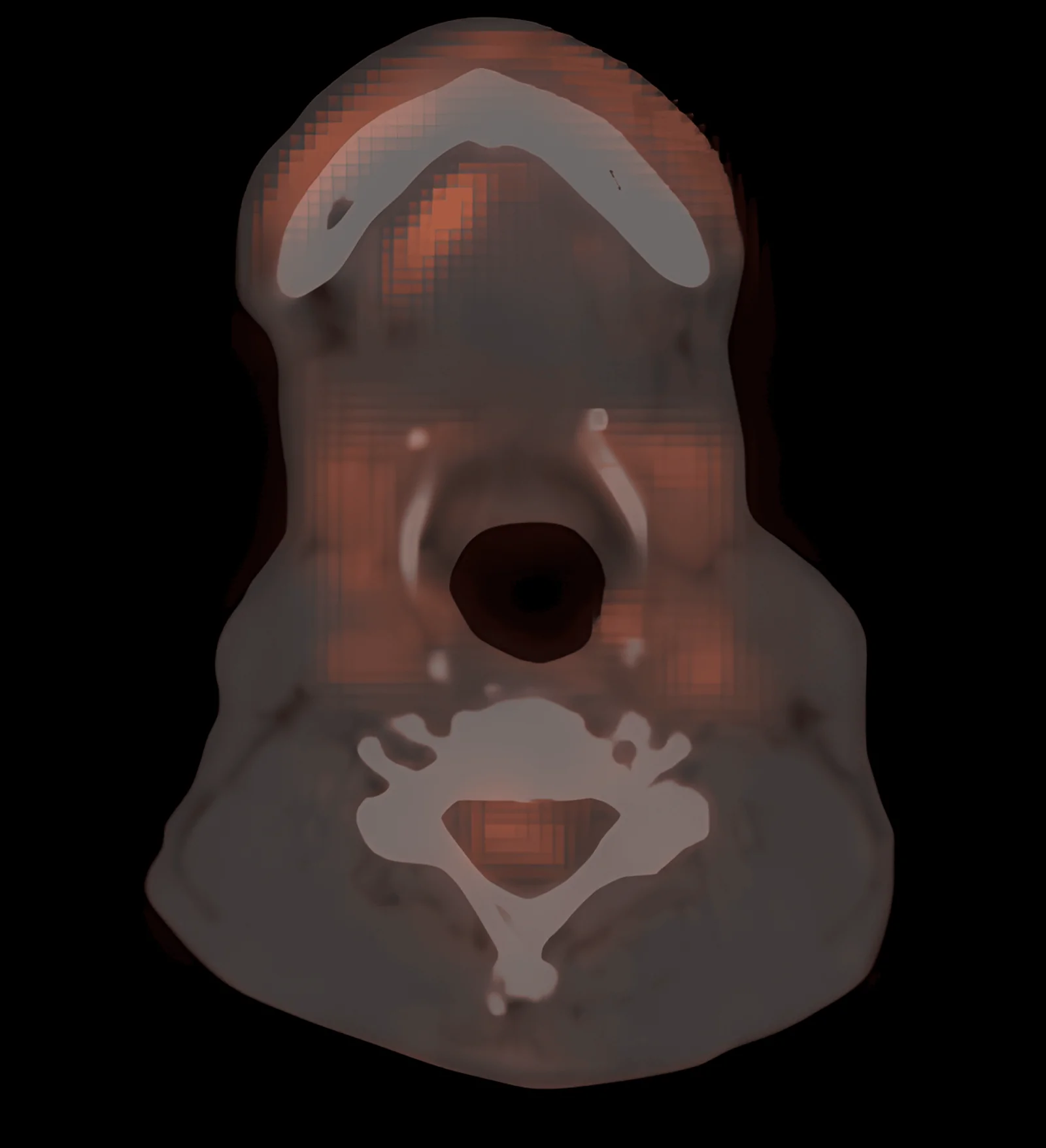
Axial 18F-fluorodeoxyglucose positron emission tommography image obtained nine months after completion of radiotherapy, showing no suspicious focal hypermetabolic activity in the floor-of-mouth region or the ipsilateral submandibular lymph node (Deauville score 2).

At the last follow-up, 18 months after completion of radiotherapy, the patient remained clinically disease-free, with no evidence of recurrence or late treatment-related toxicity.

## Discussion

Marginal zone B-cell lymphomas constitute a distinct subgroup of non-Hodgkin lymphomas characterized by an indolent clinical course and slow progression. They most commonly develop in the context of chronic immune stimulation, either related to autoimmune conditions such as Sjögren’s syndrome or rheumatoid arthritis, or to chronic infections, particularly *Helicobacter pylori* [1,2].

Oral involvement is uncommon [4], and localization to the floor of the mouth is particularly rare, with only isolated cases and small series reported in the literature [5,6]. This rarity likely contributes to diagnostic difficulties and to the limited evidence available to guide therapeutic decision-making at this specific anatomical site.

Clinically, extranodal lymphomas of the oral and maxillofacial region most often present as a localized, firm, smooth-surfaced mass, with or without ulceration, while systemic “B symptoms” such as weight loss, fever, and night sweats are uncommon [8]. Our patient similarly presented with a painless, smooth-surfaced sublingual mass that had slowly increased in size over three years, without systemic symptoms. This prolonged and minimally symptomatic presentation is consistent with the indolent clinical course of MALT lymphoma [2].

Histopathological examination of a biopsy specimen remains the cornerstone of diagnosis for extranodal marginal zone B-cell lymphoma. As MALT lymphoma lacks a specific immunohistochemical marker, diagnosis relies on the integration of morphological findings with an appropriate immunohistochemical panel. B-cell markers such as CD20 confirm the B-cell phenotype, while CD10, CD5, CD23, and cyclin D1 are particularly useful in excluding other small B-cell lymphomas [1].

In our case, the staging work-up was based on cervicofacial and thoraco-abdominopelvic CT to assess local and distant extension. PET/CT can provide additional information in selected patients with EMZL, particularly when localized treatment is planned, when clinical or laboratory findings raise suspicion of histological transformation, or to guide the selection of a biopsy site [10].

Bone marrow assessment, including aspiration and biopsy, is recommended in EMZL, particularly in non-gastric EMZL and when local treatment alone is planned, to exclude occult disseminated involvement [10]. Baseline staging should also include serological testing for hepatitis B and C viruses and HIV [10].

According to the modified Ann Arbor staging system, our case was classified as stage IIE, corresponding to extranodal involvement of the floor of the mouth associated with clinically and radiologically suspected involvement of the ipsilateral regional submandibular lymph node [10].

Although general guidelines are available for EMZL, evidence specific to oral cavity involvement remains limited. Treatment should therefore be individualized according to disease stage, tumor burden, and anatomical site, while considering the indolent nature of the disease and the need to minimize treatment-related toxicity [10,12].

For localized disease (stages I and II), involved-site radiotherapy (ISRT) represents a preferred treatment approach, providing excellent local control. Moderate radiation doses, typically 24-30 Gy, are sufficient for definitive treatment [10,11,13].

Given the indolent nature and prolonged course of low-grade non-Hodgkin lymphomas, reducing treatment-related toxicity is an important therapeutic consideration. Modern radiotherapy has addressed this issue through improved treatment techniques, reduced irradiation volumes with the adoption of ISRT and dose de-escalation strategies [11]. A very-low-dose regimen of 4 Gy delivered in two fractions can achieve meaningful responses with limited toxicity [14]. However, the randomized FoRT trial, which included follicular and marginal zone lymphomas, demonstrated inferior durable local control with 4 Gy compared with 24 Gy, despite no significant difference in overall survival. The proportion of patients with extranodal or mucosal marginal zone lymphoma was not specifically reported, limiting direct extrapolation of these findings to oral MALT lymphoma. Therefore, 24 Gy remains the standard dose when durable local control is the therapeutic objective, whereas 4 Gy may be considered in selected situations, particularly when palliation or minimization of treatment burden is prioritized [15].

Systemic therapy, including anti-CD20 immunotherapy or chemoimmunotherapy, is indicated in selected patients with EMZL, particularly in the presence of symptomatic disseminated disease, bulky disease, contraindications to radiotherapy, progression after local therapy, or suspected histological transformation [10,16]. Rituximab monotherapy is an effective option in selected patients and may provide disease control while limiting treatment-related toxicity [17]. More intensive anthracycline-containing regimens, such as R-CHOP, are generally reserved for patients with suspected or biopsy-proven histological transformation [10]. Surgery has a limited therapeutic role and is mainly used for diagnostic purposes or in selected anatomically resectable lesions [10].

In our patient’s case, the therapeutic strategy was determined through multidisciplinary discussion. Given the large tumor volume and associated regional lymph node involvement, systemic therapy was initially favored, with four cycles of rituximab monotherapy. Because the response was considered insufficient, a repeat biopsy was performed to exclude histological transformation. Although no transformation was identified, R-CHOP chemoimmunotherapy was subsequently initiated at the referring institution because the response to rituximab was considered insufficient. Given the localized and indolent nature of the disease and the absence of histological transformation, radiotherapy could have been considered earlier in the treatment course. Following a response to R-CHOP that was considered insufficient, the patient was referred to our department and received radiotherapy to the oral cavity and bilateral cervical nodal levels I-III at a dose of 24 Gy, followed by a boost to a total dose of 30 Gy to the floor-of-mouth lesion and ipsilateral submandibular lymph node. This sequential multimodal approach resulted in marked tumor regression, and subsequent PET/CT showed no suspicious hypermetabolic activity at the previously involved sites. However, the favorable outcome cannot be attributed solely to radiotherapy, as tumor regression had already occurred during systemic treatment and no comparator was available. Furthermore, the absence of baseline PET/CT precluded assessment of pretreatment FDG avidity, and the 18-month follow-up remains relatively short for an indolent lymphoma with a prolonged natural history.

MALT lymphoma is classically a slow-growing disease and is associated with a favorable prognosis, with reported five-year overall survival rates of approximately 86-95% [18,19]. Nevertheless, disease relapse may occur, and histological transformation to an aggressive lymphoma, although uncommon, represents an important adverse event [9].

MALT lymphoma International Prognostic Index (MALT-IPI) incorporates three adverse prognostic factors: age ≥70 years, advanced stage disease (Ann Arbor stage III-IV), and elevated serum lactate dehydrogenase (LDH) levels. Our patient had none of these adverse factors, being 48 years old, having stage IIE disease, and presenting with a normal baseline LDH level (231 U/L; laboratory reference range: 125-243 U/L), thus placing him in the low-risk MALT-IPI group [20].

Despite its generally favorable prognosis, the indolent and prolonged course of MALT lymphoma warrants long-term clinical surveillance to detect late relapse or histological transformation [9,10]. At the last follow-up, 18 months after completion of radiotherapy, our patient remained clinically free of recurrence, with no late treatment-related toxicity.

## Conclusions

Extranodal marginal zone B-cell lymphoma of the floor of the mouth is an exceptionally rare entity whose diagnosis requires careful histopathological evaluation supported by appropriate immunohistochemical analysis. Management should be individualized according to disease stage and clinical context. In our case, the limited anatomical response to rituximab monotherapy and subsequent R-CHOP chemoimmunotherapy illustrates the therapeutic challenges that may arise in this uncommon presentation. Following sequential systemic therapy and radiotherapy at a total dose of 30 Gy, further tumor regression was observed, and PET/CT performed nine months after radiotherapy showed no suspicious hypermetabolic activity at the previously involved sites. However, the absence of baseline PET/CT precluded formal assessment of metabolic response and attribution of the favorable outcome to any single treatment modality. Despite the generally favorable prognosis of MALT lymphoma, its prolonged clinical course and the potential for late relapse or histological transformation warrant long-term surveillance, particularly as the 18-month follow-up in this case remains relatively short for an indolent lymphoma.

## References

[1] Nakamura S, Ponzoni M (2020). Marginal zone B-cell lymphoma: lessons from Western and Eastern diagnostic approaches. Pathology.

[2] Zucca E, Bertoni F (2016). The spectrum of MALT lymphoma at different sites: biological and therapeutic relevance. Blood.

[3] Bianco L, Solla SD, Parvis G (2021). Low-dose radiotherapy for extranodal marginal zone B lymphoma of the lip: case report and literature review. Hematol Oncol Stem Cell Ther.

[4] Kaur R, Shetty D, Bagal BP (2022). Extranodal MALT lymphoma in the oral cavity: a series of three cases with review of literature. Head Neck Pathol.

[5] Hsu YT, Chiu PH, Chen YK, Chiang RP (2014). Mucosa-associated lymphoid tissue lymphoma on the mouth floor presenting with Sjögren's syndrome and giant cell tumor of spinal tendon. Kaohsiung J Med Sci.

[6] Legarrea JM, Santos ML, Rodrigues NG (2025). MALT lymphoma affecting the oral cavity: a clinical, pathologic and genetic study of MALT1 gene translocation. Oral Surg Oral Med Oral Pathol Oral Radiol.

[7] Cerhan JR, Habermann TM (2021). Epidemiology of marginal zone lymphoma. Ann Lymphoma.

[8] Triantafillidou K, Dimitrakopoulos J, Iordanidis F, Gkagkalis A (2012). Extranodal non-hodgkin lymphomas of the oral cavity and maxillofacial region: a clinical study of 58 cases and review of the literature. J Oral Maxillofac Surg.

[9] He Y, Gao C (2025). Risk and clinical implications of histological transformation in extranodal marginal zone B-cell lymphoma of mucosa-associated lymphoid tissue: a population-based analysis. Sci Rep.

[10] Eyre TA, Cwynarski K, d'Amore F (2025). Lymphomas: ESMO Clinical Practice Guideline for diagnosis, treatment and follow-up. Ann Oncol.

[11] Yahalom J, Illidge T, Specht L, Hoppe RT, Li YX, Tsang R, Wirth A (2015). Modern radiation therapy for extranodal lymphomas: field and dose guidelines from the International Lymphoma Radiation Oncology Group. Int J Radiat Oncol Biol Phys.

[12] Cheah CY, Seymour JF (2023). Marginal zone lymphoma: 2023 update on diagnosis and management. Am J Hematol.

[13] Tsang RW, Gospodarowicz MK, Pintilie M, Bezjak A, Wells W, Hodgson DC, Crump M (2001). Stage I and II MALT lymphoma: results of treatment with radiotherapy. Int J Radiat Oncol Biol Phys.

[14] Imber BS, Chau KW, Lee J (2021). Excellent response to very-low-dose radiation (4 Gy) for indolent B-cell lymphomas: is 4 Gy suitable for curable patients?. Blood Adv.

[15] Hoskin P, Popova B, Schofield O (2021). 4 Gy versus 24 Gy radiotherapy for follicular and marginal zone lymphoma (FoRT): long-term follow-up of a multicentre, randomised, phase 3, non-inferiority trial. Lancet Oncol.

[16] Zhang Q, Yan W, Li H, Peng H (2025). Advances in the pathogenesis, diagnosis, treatment, and prognosis of marginal zone lymphoma. Curr Treat Options Oncol.

[17] Mazzoni C, Argnani L, Casadei B (2025). Efficacy and safety of frontline single-agent rituximab in extranodal marginal zone lymphoma. Eur J Haematol.

[18] Yonal-Hindilerden I, Hindilerden F, Arslan S, Turan-Guzel N, Dogan IO, Nalcaci M (2016). Primary B-cell mucosa-associated lymphoid tissue lymphoma of the hard palate and parotid gland: report of one case and review of the literature. J Clin Med Res.

[19] Thieblemont C, de la Fouchardière A, Coiffier B (2003). Nongastric mucosa-associated lymphoid tissue lymphomas. Clin Lymphoma.

[20] Thieblemont C, Cascione L, Conconi A (2017). A MALT lymphoma prognostic index. Blood.

